# ﻿The adult, pupa, and larva of a new species of *Gnaptorina* Reitter, 1887 (Coleoptera, Tenebrionidae, Blaptini) from the Tibetan Plateau, with molecular phylogenetic inferences

**DOI:** 10.3897/zookeys.1190.113126

**Published:** 2024-01-23

**Authors:** Bao-Yue Ji, Xing-Tao Ma, Ji-Da Rong, Guo-Dong Ren, Zhao Pan, Xiu-Min Li

**Affiliations:** 1 Key Laboratory of Zoological Systematics and Application of Hebei Province, College of Life Sciences, Institute of Life Science and Green Development, Hebei University, Baoding 071002, China Hebei University Hebei China

**Keywords:** Beetle, China, DNA sequence, morphological description

## Abstract

The adult, pupa and larva of a new species, Gnaptorina (Gnaptorina) lhorongica Li, **sp. nov.**, from northeastern Xizang, China are described and illustrated. The species was identified using molecular phylogenetic analyses based on three mitochondrial fragments and one nuclear gene fragment (*COI*, *Cytb*, *16S*, and *28S*-D2). The taxonomic status of the new species is confirmed using a combination of molecular and morphological datasets. This study provides valuable molecular and morphological data for phylogenetic studies of the tribe Blaptini.

## ﻿Introduction

The genus *Gnaptorina* Reitter, 1887 belongs to the subtribe Gnaptorinina Medvedev, 2001 of the tribe Blaptini Leach, 1815 of the subfamily Blaptinae Leach, 1815 (Kamiński et al. 2021). The Gnaptorinina is a species-rich subtribe in Blaptini, consisting of 11 genera. Larval and pupal morphology is important for understanding the systematics of different groups of Coleoptera, and it has been used to support the close relationships of genera or subtribes (Grebennikov and Scholtz 2004; Lawrence et al. 2011; Chigray 2019; Kamiński et al. 2019). To date, the morphology of immature stages of seven genera and 40 species are known within Blaptini: *Blaps* Fabricius, 1775 (larvae of 25 spp. and pupae of 10 spp.), *Prosodes* Eschscholtz, 1829 (larvae of five spp.), *Dila* Fischer von Waldheim, 1844 (larvae of two spp.), *Nalepa* Reitter, 1887 (larvae of two spp.), *Gnaptorina* Reitter, 1887 (larvae of two spp.), *Agnaptoria* Reitter, 1887 (larvae of two spp.) and *Itagonia* Reitter, 1887 (larva of one sp.) (Yu et al. 1993a, 1993b, 1996, 1999a, 1999b, 2000; Ren et al. 2000; Zhang et al. 2000, 2005; Yu and Zhang 2004, 2005; Zhao et al. 2009; Zhu and Ren 2014; Li et al. 2022, 2023; Tang et al. 2023).

*Gnaptorina* is the third largest genus of the subtribe Gnaptorinina with 38 described species (Medvedev 2009; Shi 2013; Ren et al. 2016; Li et al. 2019; Bai et al. 2020). All *Gnaptorina* species have been recorded in China, except for *G.sikkimensis* Kaszab, 1965, which is endemic to northern India, and *G.brucei* Blair, 1923, which occurs in Nepal and northern India. However, the larval morphology of only two species (*G.felicitana* Reitter, 1887 and *G.cylindricollis* Reitter, 1889) have been described (Yu et al. 1996; Zhu and Ren 2014), and pupal morphology is still unknown due to the lack of specimens.

In this study, the adults, pupa and larva of a new *Gnaptorina* species are described based on morphological and molecular evidence. In addition, we construct a molecular phylogeny for the genus and apply it to verify the taxonomic status of the new species.

## ﻿Materials and methods

### ﻿Morphological examination

In total, 64 adults, two larvae, and one pupa of the new species were examined for this study and deposited at the Museum of Hebei University, Baoding, China (**MHBU**).

The photos were taken with three imaging systems: (a) Canon EOS 5D Mark III (Canon Inc., Tokyo, Japan) connected to a Laowa FF 100 mm F2.8 CA-Dreamer Macro 2× or Laowa FF 25 mm F2.8 Ultra Macro 2.5–5× (Anhui Changgeng Optics Technology Co., Hefei, China); (b) a Leica M205A stereomicroscope equipped with a Leica DFC450 camera (Leica Microsystems, Singapore, Singapore), which was controlled using the Leica application suite v. 4.3; (c) JVC KY-F75U (JVC Kenwood, Long Beach, CA, USA) digital camera attached to a Leica Z16 APO dissecting microscope (Leica Microsystems, Buffalo Grove, IL, USA) with an apochromatic zoom objective and motor focus drive, using a Syncroscopy Auto-Montage System (Synoptics, Cambridge, UK) and software. Multiple images were used to construct the final figures. Images were illuminated with either an LED ring light attached to the end of the microscope column, with incidental light filtered to reduce glare, or by a gooseneck illuminator with bifurcating fiberoptics; image stacks were white-balance corrected using the system software (Synoptics, Cambridge, UK). Montaged images were edited using Adobe Photoshop v. 22.1.0 to form the final figure plates.

Label data are presented verbatim. A slash (/) separates text on different lines of label. A double slash (//) separates text on separate lines of a label; authors’ remarks are enclosed in brackets “[]”.

### ﻿Taxon sampling, DNA extraction, PCR amplification, and sequencing

Larval specimens were collected in the field together with adults from the Tibetan Plateau, China. To correlate the different stages, the molecular data were collected from six individuals (four adults, one larva, and one pupa).

DNA was extracted from the pygopod tissues of the larva and pupa, and from the leg muscle tissue of the adults using the Insect DNA isolation Kit (BIOMIGA, Dalian, China) following the manufacturer’s protocols. The DNA extracted was stored at –20 °C. Fragments of three mitochondrial markers (cytochrome oxidase subunit I, *COI*; cytochrome b, *Cytb*; *16S* ribosomal DNA, *16S*), and one nuclear marker (*28S* ribosomal DNA domain D2, *28S*-D2) were amplified and sequenced. The primers and the annealing temperatures are shown in Table 1.

**Table 1.** Primer sequences for PCR.

Gene	Primer (forward/reverse)	Sequence (forward and reverse) 5′→3′	PCR conditions (annealing)	References
*COI*	F 2183	CAACATTTATTTTGATTTTTTGG	50 °C	Monteiro and Pierce 2001
	R 3014	TCCAATGCACTAATCTGCCATATTA		
*Cytb*	F revcb2h	TGAGGACAAATATCATTTTGAGGW	50 °C	Simmons and Weller 2001
	R rebcbj	TCAGGTCGAGCTCCAATTCATGT		
*16S*	F 13398	CGCCTGTTTATCAAAAACAT	50 °C	Simon et al. 1994
	R 12887	CCGGTCTGAACTCAGATCAT		
*28S-D2*	F 3665	AGAGAGAGTTCAAGAGTACGTG	58 °C	Belshaw and Quicke 1997
	R 4068	TTGGTCCGTGTTTCAAGACGGG		

The profile of the PCR amplification consisted of an initial denaturation step at 94 °C for 4 min, 35 cycles of denaturation at 94 °C for 1 min, annealing for 45 s, an extension at 72 °C for 1 min, and a final 8 min extension step at 72 °C. PCR was performed using TaKaRa Ex Taq (TaKaRa, Dalian, China). PCR products were subsequently checked by 1% agarose gel electrophoresis and sequencing was performed at General Biol Co. (Anhui, China). Altogether, all molecular data were collected from 82 individuals (80 adults, one larva, and one pupa); 89 new sequences from 26 individuals of nine species were generated, and 211 sequences were previously published (Li et al. 2021). We used previously published sequences of four Platyscelidini Lacordaire, 1859 species as the outgroup, which has been considered a close relative of the tribe Blaptini (Kamiński et al. 2021). Detailed information for all the samples used in this study is provided in Suppl. material 1.

### ﻿Phylogenetic analyses

Phylogenetic analyses were based on the concatenated dataset under the maximum likelihood (ML) criterion in IQ-TREE v. 1.6.6 (Nguyen et al. 2015), as implemented in the dedicated IQ-TREE web server (http://iqtree.cibiv.univie.ac.at/, accessed 2023-6-1). The ML tree was inferred under an edge-linked partition model for 5000 ultrafast bootstraps (1000 replicates) (Minh et al. 2013). The consensus phylogenetic tree was visualized in Figtree v. 1.4.4 (http://tree.bio.ed.ac.uk/software/figtree, accessed 2023-6-1).

## ﻿Results

### ﻿Morphological study and diagnosis

#### ﻿ Gnaptorina (Gnaptorina) lhorongica

Taxon classificationAnimaliaColeopteraTenebrionidae

Li sp. nov.

A954A7EF-E446-51F8-B4D9-E6CAE947B79C

https://zoobank.org/0CB92107-F71F-48C0-98FD-9EF1FA9CA7F5

**Figure 1.**
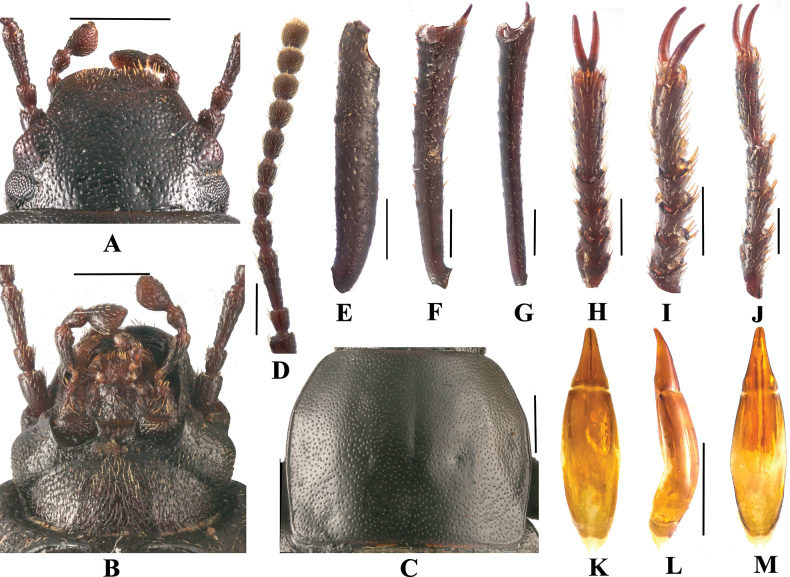
Gnaptorina (Gnaptorina) lhorongica Li, sp. nov. Holotype **A** head, dorsal view **B** head, ventral view **C** pronotum **D** antenna **E** protibia **F** mesotibia **G** metatibia **H** protarsus **I** mesotarsus **J** metatarsus **K** aedeagus, dorsal view **L** aedeagus, lateral view **M** aedeagus, ventral view. Scale bars: 1.0 mm (**A–C, K–M**); 0.5 mm (**D–J**).

Figs 1 , 3 , 5 , 6 , 7

##### Type locality.

Lajiu Township, Lhorong County, Xizang.

##### Type materials

**(Adults). *Holotype***: China • ♂//西藏洛隆腊久乡 [Lajiu Township, Lhorong County, Xizang]/ 30°28.714′N, 95°53.593′E/ Alt. 4680 m /labeled 30.Jul. 2019/ 任国栋, 李亚林, 白兴龙 [Guo-Dong Ren, Ya-lin Li & Xing-Long Bai leg.]. ***Paratypes***: 4♂6♀// 西藏洛隆腊久乡 [Lajiu Township, Lhorong County, Xizang]/ 30°28.714′N, 95°53.593′E/ Alt. 4680 m/ labeled 30. Jul. 2019/ 任国栋, 李亚林, 白兴龙 [Guo-Dong Ren, Ya-lin Li & Xing-Long Bai leg.]; 6♂4♀// 西藏洛隆腊久乡[Lajiu Township, Lhorong County, Xizang]/ 30°25.203′N, 96°5.950′E/ Alt. 3910 m/ labeled 30.Jul. 2019/ 任国栋,李亚林, 白兴龙 [Guo-Dong Ren, Ya-lin Li & Xing-Long Bai leg.]; 6♂8♀// same data as holotype; 10♂8♀// 西藏洛隆孜托镇 [Zituo Township, Lhorong County, Xizang]/ 30°32.515′N, 95°46.774′E/ Alt. 4031 m/ labeled 30.Jul. 2019/ 任国栋, 李亚林, 白兴龙 [Guo-Dong Ren, Ya-lin Li & Xing-Long Bai leg.]; 3♂8♀// 西藏洛隆达翁拉山 [Daonla mountain, Lhorong County, Xizang]/ 30°46.204′N, 95°33.758′E/ Alt. 3854 m/ labeled 11.Jul. 2015/ 任国栋, 白兴龙 [Guo-Dong Ren & Xing-Long Bai leg.].

##### Other examined materials.

**Larva.** 2 ex. // 西藏洛隆腊久乡 [Lajiu Township, Lhorong County, Xizang]/ 30°28.714′N, 95°53.593′E/ Alt. 4680 m/ labeled 30.Jul. 2019/ 任国栋, 李亚林, 白兴龙 [Guo-Dong Ren, Ya-lin Li & Xing-Long Bai leg.].

**Pupa.** 1♂// 西藏洛隆腊久乡 [Lajiu Township, Lhorong County, Xizang]/ 30°28.714′N, 95°53.593′E/ Alt. 4680 m/ labeled 30.Jul. 2019/ 任国栋, 李亚林, 白兴龙 [Guo-Dong Ren, Ya-lin Li & Xing-Long Bai leg.].

##### Description of adult.

Body length 10.5–11.2mm, width 5.8–6.0 mm; body shiny, black; antennae, palpi, and tarsi brown.

**Figure 2.**
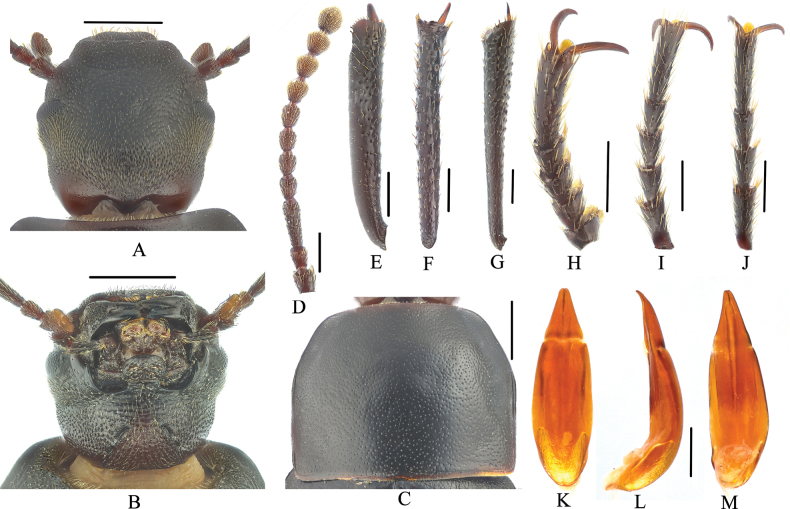
Gnaptorina (Gnaptorina) dongdashanensis Shi, 2013 **A** head, dorsal view **B** head, ventral view **C** pronotum **D** antenna **E** protibia **F** mesotibia **G** metatibia **H** protarsus **I** mesotarsus **J** metatarsus **K** aedeagus, dorsal view **L** aedeagus, lateral view **M** aedeagus, ventral view. Scale bars: 1.0 mm (**A–C**); 0.5 mm (**D–M**).

**Male** (Figs 1A–L, 2). ***Head***: (Fig. 1A). Anterior margin of clypeus weakly sinuate. Lateral margin of head with distinct emargination between epistome and genae. Head widest at eye level. Lateral margin of head with pair of projections between antennal base and oculus, brownish red. Genal margin arcuately converging before eyes. Eyes barely protruding beyond contour of head. Vertex flat or slightly convex, with uniform punctures. Antennae (Fig. 1D) slender and long, reaching beyond pronotal base when posteriorly extended, antennomeres VIII–X oval, XI spindle-shaped. Length (width) ratio of antennomeres II–XI as follows: 10.0(8.0): 26.0(8.0): 13.0(8.0): 12.0(8.0): 12.0(8.0): 13.0(9.0): 12.0(10.0): 11.0(10.0): 11.0(11.0): 11.0(11.0).

***Prothorax*.** Pronotum (Fig. 1C) transverse, 1.31–1.33 times as wide as long. Broadest at middle, 1.80–1.85 times as wide as head. Ratio of width at anterior margin to its maximum width and base 23: 37: 34. Anterior margin straight or weakly sinuate, laterally beaded. Lateral margins weakly wider from base to middle and narrowing toward anterior angles arcuately. Anterior angles obtuse, rounded apically; posterior angles weakly obtuse, nearly rectangular. Disc convex, with shallow and circular depressions laterally before base, surface with dense, fine punctures. Prothoracic hypomeron weakly concave, with longitudinal wrinkles and sparse and minute granules. Prosternal process steeply sloping behind procoxae and forming wide and flat prominence at end of declivity.

***Pterothorax*.** Elytra widely oval and convex, 1.12–1.26 times as long as wide, 1.61–1.77 times as wide as pronotum, widest before middle. Surface with shallow, fine, sparse punctures and irregular, short wrinkles. Lateral margins reaching sutural elytral angle, visible dorsally in anterior third and apex. Surface of epipleura smooth, with shallow wrinkles.

***Legs*** (Fig. 1E–J) slender and long. Profemora with obtuse tooth. Protibiae straight, distal apical spur obviously shorter than protarsomere 1, lower spur shorter; ventral surface of protarsomeres 1 and 2 with hairy brush. Mesotibiae weakly curved; ventral surface of mesotarsomere 1 with hairy brush at apex. Metatibiae straight, regularly widening apicad. Ratio of length(width) pro-, meso-, and metatibiae: 80.0(15.0): 95.0(17.0): 138.0(20.0), that of metatarsomeres I–IV as follows: 19.0(11.0): 20.0(10.9): 17.0(9.6):37.0(9.8).

***Aedeagus*.** (Fig. 1K–M) 2.20 mm long and 0.58 mm wide. Parameres 0.53 mm long and 0.37 mm wide, conical, widest at base, with outer margins weakly sinuate near middle, and regularly narrowing towards apex laterally in dorsal view; dorsal side nearly straight, slightly curved to ventral side apically in lateral view.

**Figure 3.**
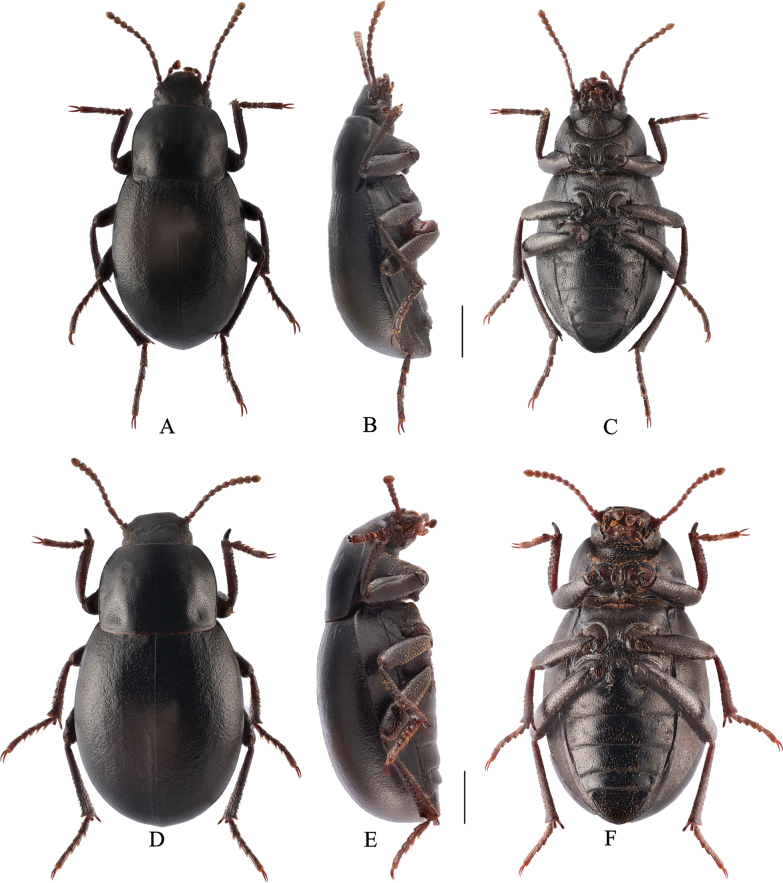
Gnaptorina (Gnaptorina) lhorongica Li, sp. nov. **A–C** male, holotype **D–F** female, paratype **A**, **D** dorsal view **B, E** lateral view **C, F** ventral view. Scale bars: 2.0 mm.

**Female** (Fig. 3). Body length 11.2–11.5mm, width 5.9–6.2 mm. Body wider than male. Head 1.14 times as wide as interocular distance. Pronotum 1.28–1.30 times as wide as long, widest in middle, lateral margins subparallel from base to middle, then narrowing toward anterior angles arcuated. Elytra more convex, 1.13 times as long as wide. Antennae shorter than in male. Upper spur of protibiae wide and flat; lower spur fine and pointed. Ventral surface of pro and mesotarsomeres I–IV with hairy brush.

##### Etymology.

Named after the county of Lhorong, where the type locality is located.

##### Distribution.

China: Xizang.

##### Diagnosis.

**Figure 4.**
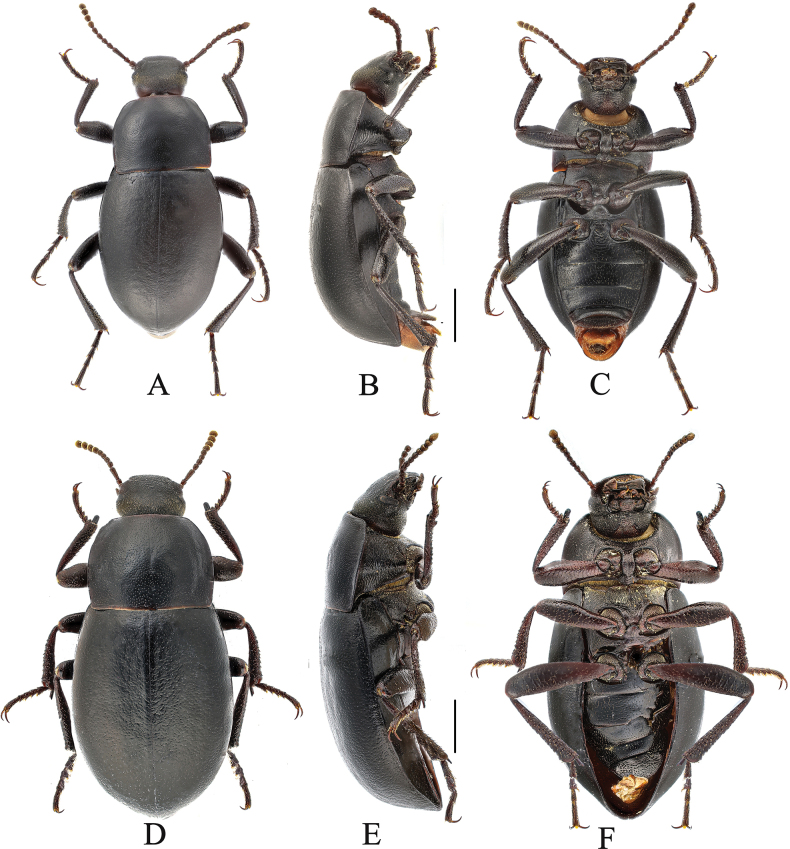
Gnaptorina (Gnaptorina) dongdashanensis Shi, 2013 **A–C** male **D–F** female **A**, **D** dorsal view **B, E** lateral view **C, F** ventral view. Scale bars: 2.0 mm.

This new species is morphologically similar to G. (G.) dongdashanensis Shi, 2013 but can be distinguished from it by the following male character states: (1) genal margin arcuately converging before eyes (genal margin parallel before eyes in *G.dongdashanensis*); (2) antennomeres IV–VII long and cylindrical (antennomeres IV–VII nearly spherical in *G.dongdashanensis*); (4) elytral widely oval (elytral elongate-oval in *G.dongdashanensis*). (Figs 2, 4)

##### Description of larva.

**Figure 5.**
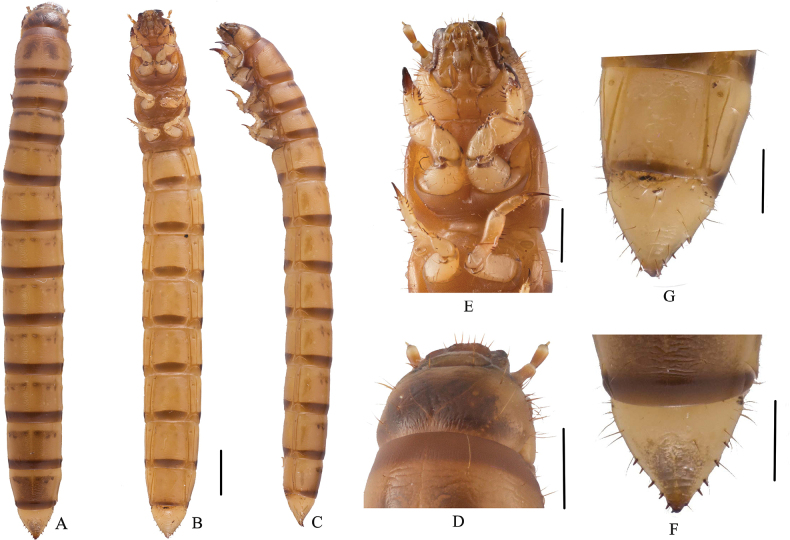
Larva of Gnaptorina (Gnaptorina) lhorongica Li, sp. nov. **A–C** habitus **A** dorsal view **B** ventral view **C** lateral view **D** head, dorsal view **E** head, fore foot, and mesoleg, in ventral view **F** pygopods, in dorsal view **G** pygopods, in ventral view. Scale bars: 2 mm (**A–C**); 1 mm (**D–G**).

***Body*.** (Fig. 5A–C) Mature larvae length 23.0–25.0mm, width 2.5–3.0mm. Body subcylindrical; 9^th^ abdominal tergite conical and urogomphi not sharp; body brownish yellow, shiny; body wall ossified; median line obvious on first four segments; pairs of setae grow on each tergite; terga I–VIII with four pairs of long setae, two pairs anterior and two pairs posterior.

**Figure 6.**
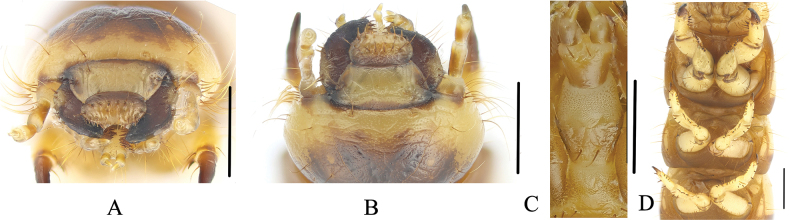
Larva of Gnaptorina (Gnaptorina) lhorongica Li, sp. nov. **A** head **B** labrum and clypeus, in dorsal view **C** labium **D** legs. Scale bars: 1 mm (**A, B, D**); 0.5 mm (**C**).

***Head*** (Figs 5D, E, 6A–C). Prognathous slightly narrower than width of prothorax, slightly convex dorsally, and sides rounded (Fig. 5D). Labrum transverse; apical part with six setae. Mandibles well developed; left and right symmetrical; each mandible with two pair of setae; clypeus transverse, trapezoidal, left and right marginal sides with two pairs of short setae (Fig. 6A, B). Epicranial stem Y-shaped (Fig. 5E); frons and epicranial plate slightly convex, lateral margin with densely long setae, frons with four pairs of setae. Maxillary palps three-segmented, cylindrical, and conical at apex; I widest, II longest, I as long as III (Fig. 5D, E). Labial palps (Fig. 5D) two-segmented, short; II conical. Mentum convex, U-shaped, base of mentum straight, prementum with two long setae, mentum with two long setae on posterior margin, submentum with eight setae on mid-posterior part (Fig. 6C). Antennae well developed, three-segmented, dome-like at apex; segment I wider and as long as II; segment III shortest and narrowest.

***Thorax*** (Fig. 5A–C). Thoracic segmentation C-shaped in dorsal view, parallel-sided, widest at middle, with transverse plicae. Each thoracic tergum with two pairs of elongate setae on anterior and posterior margins. Anterior and posterior border of prothorax with brown longitudinal stripes, with pair of irregular brown spots on tergite, pair of brown bands on top dorsal margins of mesothorax and metathorax; two pairs of irregular brown spots on tergites IV–XI. Pronotum longest, about twice as long as meso- and metanotum, mesonotum shortest.

***Legs*** (Figs 5E, 6D). Legs well developed. Prothoracic leg noticeably stronger, longer, and thicker than meso- and metathoracic legs; profemur and protibia with a row of spines and denser long setae (Fig. 5E). Protarsungulus strongly sclerotized, sharp, claw-like; one strong, long seta on inner side of the base of the protarsungulus, and one strong, short spine on posterior outer side. Profemora and protibiae gradually narrowing towards apex; inner margin setal formula of foreleg 4–5(3): 6(5): 2(2); outer margin of tibiae with two setae; outer margin of femora with two setae; trochanter with three setae. Mesotarsus with one short, broad spine at base; inner margin setal formula of mesothoracic leg 4(3): 5(3): 2(2); outer margin of tibiae with two short spines; outer margin of femora with two spines; outer margin of trochanters with three setae. Metatarsus with one short, broad spine at base, the inner margin setal formula of metathoracic leg 4(3): 4(3): 2(2), outer margin of tibiae with two short spines, outer margin of femora with two to three spines, outer margin of trochanters with two setae. Profemora about half length of protibia; meso- and metathoracic legs moderately shorter than prothoracic one, tarsungulus highly ossified, hooked, with a row of spines and sparse setae.

***Abdomen*** (Fig. 5A–C). Approximately 3.6 times as long as thorax; segments I–VIII subcylindrical, with transverses plicae, faintly rugose, and with sparse elongate setae ventrally; tergum of IX 0.75 times as long as tergum VIII, distinctly narrower than tergum VIII; with a row of short spines each side (five spines on left, four spines on right); last segment conical in dorsal view, surface of convex disc with sparse long setae in ventral view; urogomphi suddenly upturned to apex in lateral view, apex truncated, with two thorn-like processes.

***Spiracles*** (Fig. 5B). Pair of circular thoracic spiracles, situated ventrolaterally on anterolateral margins of terga I–VIII.

##### Diagnosis of larva.

The larva of new species is morphologically very similar to G. (G.) cylindricollis Reitter, 1889, but can be distinguished from it by the following characters: (1) lateral margins of the head with dense long setae (lateral margins of the head with sparse long setae in *G.cylindricollis*); (2) mentum with two long setae on the side of the posterior margin and submentum with eight setae on located in the middle, posteriorly (mentum with four long setae on the side of the posterior margin and submentum with five setae in the posterior part of the center in *G.cylindricollis*).

The larva of new species is morphologically very similar to G. (G.) felicitana but can be distinguished from the latter by the following characters: (1) lateral margins of the head with dense, long setae (G. (G.) felicitana with sparse, long setae); (2) terga I–VIII with four pairs of long setae, two anterior pairs and two posterior pairs (G. (G.) felicitana with six pairs of long setae, three anterior pairs and three posterior pairs); (3) frons with eight setae, two on upper margin, six on apex (G. (G.) felicitana with eight setae, two on upper margin, two at center, and four at posterior margin in); (4) apex of antennomere III without long setae (G. (G.) felicitana with one long seta at the apex); (5) side posterior margin of mentum with two long setae and middle of submentum with eight setae posteriorly (G. (G.) felicitana with mentum and submentum both with 10 setae).

##### Description of pupa.

**Figure 7.**
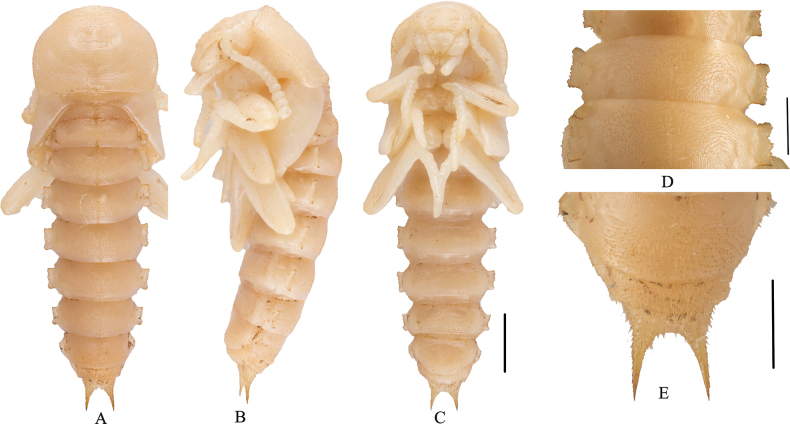
Pupa of Gnaptorina (Gnaptorina) lhorongica Li, sp. nov. **A–C** habitus **A** dorsal view **B** ventral view **C** lateral view **D** lateral process of abdominal terga, in dorsal view **E** urogomphy, in dorsal view. Scale bars: 2 mm (**A–C**); 1 mm (**D, E**).

***Body*** (Fig. 7A–C). Length 13.5 mm, width 3.6 mm. Body moderately elongated, slightly flattened, tapering towards posterior and with pronotum widest, creamy white.

***Head*** (Figs 7B, C). Invisible in dorsal view. Smooth, with transverse wrinkles. Head bending towards underside of prothorax, slightly elevated at center of head, with sparse, short setae on margins. Labrum and mandible smooth, covered with sparse, short setae; anterior margin of clypeus straight, sides weakly curved. Antennae rod-shaped, gradually thickened. Maxillary and labial palpi visible clearly.

***Thorax*** (Fig. 7A–C). Pronotum semicircular and with posterior margin straight, about 1.6 times as long as wide, widest in middle. Pronotum depressed medially, with transverse plicae, with sparse short setae on top to anterior margin and sparse short setae lateral margin in dorsal view. Elytra narrowed proximally to form alaria, surface smooth but with sparse short setae.

***Legs*** (Fig. 7B, C). Legs similar to adults. Femora and tibiae with minute setae; tarsi glabrous, extended anteriorly. Fore leg shortest; hind leg longest.

***Abdomen*** (Fig. 7A, C, D). Abdomen nine-segmented, with distinct midline. Terga broad, convex, covered with minute, short setae. Terga I–VI flanked by outwardly projecting, plate-like lateral processes; with sparse, short setae. Lateral process of tergum VII triangular; lateral process of tergum VIII less developed. Tergum IX with a pair of elongate urogomphi at apex 1.13 mm long. (Fig. 7E).

### ﻿Phylogenetic relationships

**Figure 8.**
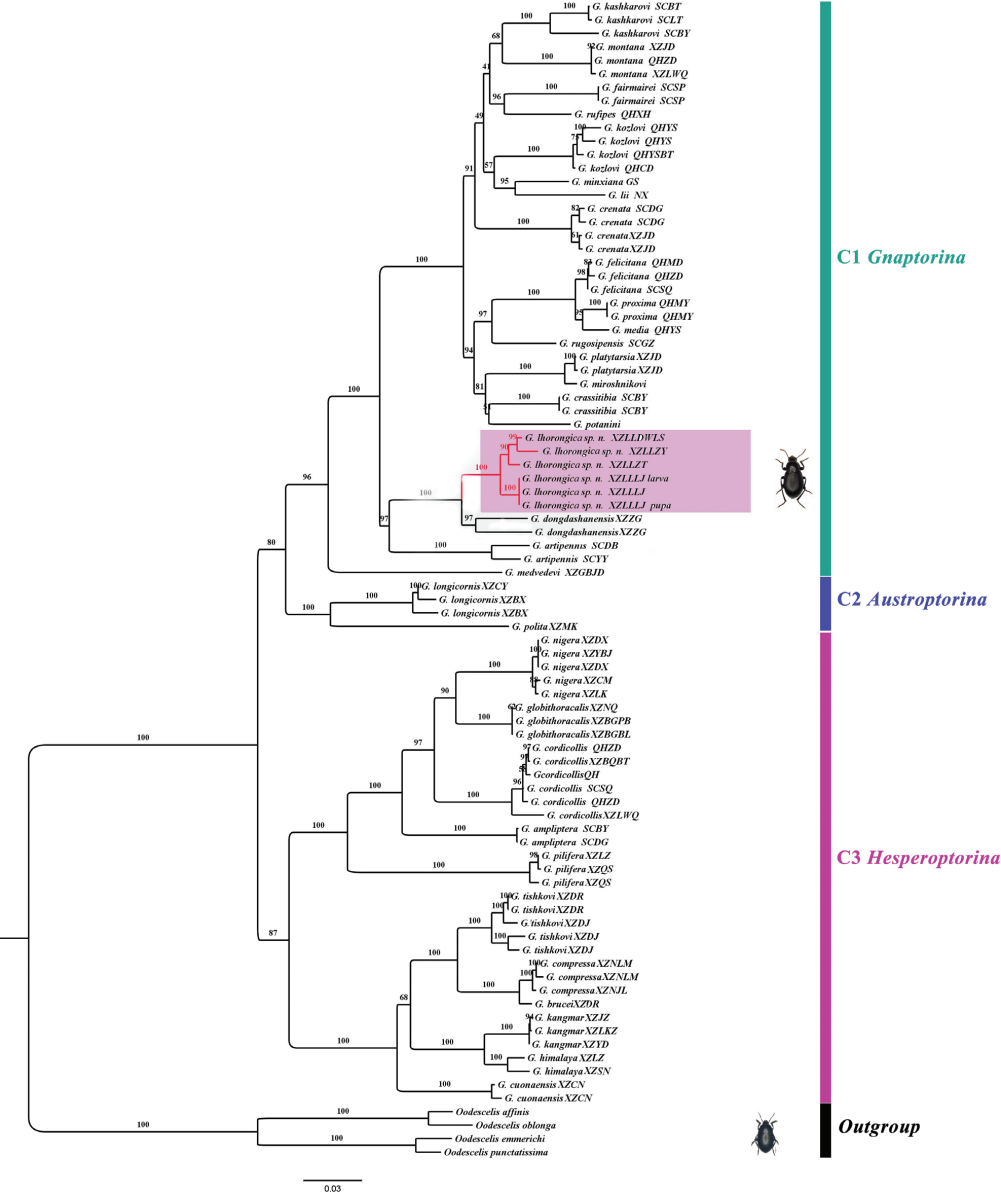
Maximum-likelihood phylogenetic tree based on 2321 bp of mitochondrial and nuclear DNA sequences (*COI*, *Cytb*, *16S*, and *28S*-D2) within the genus *Gnaptorina*. Support for each node is represented by ultrafast bootstrap values (uBV).

The final, concatenated dataset was 2321-bp long, including 300 sequences from 82 specimens of 32 described species and six specimens of the new species (*COI*, 648 bp; *Cytb*, 579 bp; *16S*, 496 bp; *28S-D2*, 443 bp). IQ-TREE analyses yielded a topology, and the preliminary phylogenetic relationship was hypothesized for the genus *Gnaptorina* (Fig. 8).

The ML tree revealed that there was a reasonable correlation of membership of these major clades. The monophy of the subgenera was well supported overall. The individuals of *Gnaptorina* were grouped into three well-supported clades: clade C1 (*Gnaptorina*, uBV = 96), clade C2 (*Austroptorina*, uBV = 100) and clade C3 (*Hesperoptorina*, uBV = 87). The taxonomic status of the new species is confirmed by phylogenetic relationships and morphological evidence: G. (G.) lhorongica Li, sp. nov. The adult, pupa, and larva cluster into a single well-supported clade (uBV = 100). Based on the above results, the larval and pupal samples are confirmed as adult stages of G. (G.) lhorongica Li, sp. nov. The molecular analyses also indicate that the new species is a closely related and sister to G. (G.) dongdashanensis.

## ﻿Discussion

The adults, pupa, and larva of *G.lhorongica* Li, sp. nov. were collected in the field; hence, it was rather difficult to judge the larval developmental stage. The larva used for the description above was inferred to be in its final instar stage based on previous research on the larval biology of the Blaptini.

The genus *Gnaptorina* comprises 38 species (Bai et al. 2020), which are mostly distributed in high elevations on the Tibetan Plateau (Li et al. 2021). It is difficult to obtain larvae and pupae by rearing adults, because habitat conditions of *Gnaptorina* in the wild are not possible to replicate. To date, the larval morphology of only *G.felicitana* and *G.cylindricollis* have been described and pupal morphology in *Gnaptorina* has not been updated before the present study (Yu et al. 1996; Zhu and Ren 2014). Our present description of the pupa for G. (G.) lhorongica Li, sp. nov . is the first for the genus. Thus, it is currently impossible to provide a generic diagnosis for pupae of *Gnaptorina*, and our understanding of the morphological diversity within *Gnaptorina* is clearly in its infancy; it would be premature to attempt a diagnosis for larvae and pupae of the genus. We hope to discover additional larvae and pupae and associate them with their respective adults by rearing or molecular analysis. Only with the discovery of larvae and pupae for additional *Gnaptorina* species may we offer sound generic diagnoses and more robust hypotheses of relationships. Meanwhile, the molecular database of the genus *Gnaptorina* offers strong support for studying unknown species at any stage of development.

## Supplementary Material

XML Treatment for Gnaptorina (Gnaptorina) lhorongica

## ﻿Additional information

### Author ORCIDs

Bao-Yue Ji https://orcid.org/0000-0002-9250-5481

Xing-Tao Ma https://orcid.org/0009-0000-4075-7292

Ji-Da Rong https://orcid.org/0009-0008-3207-5302

Guo-Dong Ren https://orcid.org/0000-0001-5808-9122

Zhao-Pan https://orcid.org/0000-0001-7798-0009

Xiu-Min Li https://orcid.org/0000-0003-0575-1869

### Data availability

All of the data that support the findings of this study are available in the main text or Supplementary Information.

## Supplementary materials

Supplementary material 1 List of specimens used in this study with the corresponding accession number Data type docx https://binary.pensoft.net/file/972450This dataset is made available under the Open Database License (http://opendatacommons.org/licenses/odbl/1.0/). The Open Database License (ODbL) is a license agreement intended to allow users to freely share, modify, and use this Dataset while maintaining this same freedom for others, provided that the original source and author(s) are credited.Xiu-Min Li

## References

[B1] Bai XL Li XM Ren GD (2020) Description of a new subgenus and four new species of *Gnaptorina* Reitter, 1887 (Coleoptera: Tenebrionidae: Blaptini) from China. Zootaxa 4809(1): 165–176. 10.11646/zootaxa.4809.1.10 33055955

[B2] Belshaw R Quicke DLJ (1997) A molecular phylogeny of the Aphidiinae (Hymenoptera: Braconidae). Molecular Phylogenetics and Evolution 7(3): 281–293. 10.1006/mpev.1996.0400 9187088

[B3] Chigray IA (2019) A new genus and species of darkling beetles of the tribe Blaptini (Coleoptera: Tenebrionidae) from Afghanistan and taxonomic changes in the tribe. Entomological Review 99(7): 914–923. 10.1134/S0013873819070054

[B4] Grebennikov VV Scholtz CH (2004) The basal phylogeny of Scarabaeoidea (Insecta: Coleoptera) inferred from larval morphology. Invertebrate Systematics 18(3): 321–348. 10.1071/IS03013

[B5] Kamiński MJ Lumen R Kubicz M Steiner W Iwan D (2019) Immature stages of beetles representing the ‘Opatrinoid’ clade (Coleoptera: Tenebrionidae): an overview of current knowledge of the larval morphology and some resulting taxonomic notes on Blapstinina. Zoomorphology 138(4): 349–370. 10.1007/s00435-019-00443-7

[B6] Kamiński MJ Lumen R Kanda K Iwan D Johnston MA Kergoat GJ Bouchard P Bai XL Li XM Ren GD Smith AD (2021) Reevaluation of Blapimorpha and Opatrinae: addressing a major phylogeny-classification gap in darkling beetles (Coleoptera: Tenebrionidae: Blaptinae). Systematic Entomology 46(1): 140–156. 10.1111/syen.12453

[B7] Lawrence JF Seago AE Newton AF Thayer MK Marvaldi AE Slipinski A (2011) Phylogeny of the Coleoptera based on morphological characters of adults and larvae. Annales Zoologici 61(1): 1–217. 10.3161/000345411X576725

[B8] Li XM Bai XL Ren GD (2019) Two new species of the genus *Gnaptorina* Reitter from the Hengduan Mountains, China (Coleoptera: Tenebrionidae: Blaptini). Zootaxa 4695(1): 83–89. 10.11646/zootaxa.4695.1.7 31719368

[B9] Li XM Bai XL Kergoat GJ Pan Z Ren GD (2021) Phylogenetics, historical biogeography and molecular species delimitation of *Gnaptorina* Reitter (Coleoptera: Tenebrionidae: Blaptini). Systematic Entomology 46(1): 239–251. 10.1111/syen.12459

[B10] Li XM Tian J Fan JJ Ren GD (2022) Systematic review of the genus *Nalepa* Reitter, 1887 (Coleoptera, Tenebrionidae, Blaptinae, Blaptini) from the Tibetan Plateau, with description of six new species and two larvae. Insects 13(7): 598. 10.3390/insects13070598 35886774 PMC9316563

[B11] Li XM Ji BY Tian J Ren GD (2023) The adult and larva of a new species of the genus *Dila* (Coleoptera, Blaptinae, Blaptini) from Himalayas, with molecular phylogenetic inferences of related genera of the Blaptini. Insects 14(3): 284. 10.3390/insects14030284 36975968 PMC10099737

[B12] Medvedev GS (2001) Evolution and system of darkling beetles of the tribe Blaptini (Coleoptera, Tenebrionidae). Meetings in memory of N.A. Cholodkovsky. Iss. 53., St. Petersburg, 332 pp.

[B13] Medvedev GS (2009) Composition of the genera *Gnaptorina* Reitter and *Pseudognaptorina* Kaszab of the tribe Blaptini (Coleoptera, Tenebrionidae). Entomologicheskoe Obozrenie 88: 416–429. [in Russian, English translation: Entomological Review 89: 451–461]. 10.1134/S0013873809040095

[B14] Minh BQ Nguyen MAT Von Haeseler A (2013) Ultrafast approximation for phylogenetic bootstrap. Molecular Biology and Evolution 30(5): 1188–1195. 10.1093/molbev/mst024 23418397 PMC3670741

[B15] Monteiro A Pierce NE (2001) Phylogeny of *Bicyclus* (Lepidoptera:Nymphalidae) inferred from *COI*, *COII* and EF-1α gene sequences. Molecular Phylogenetics and Evolution 18(2): 264–281. 10.1006/mpev.2000.0872 11161761

[B16] Nguyen LT Schmidt HA Von Haeseler A Minh BQ (2015) IQ-TREE: A fast and effective stochastic algorithm for estimating maximum-likelihood phylogenies. Molecular Biology and Evolution 32(1): 268–274. 10.1093/molbev/msu300 25371430 PMC4271533

[B17] Ren GD Yu YZ Yang XJ (2000) A list of the known darkling beetles-larvae (Coleoptera: Tenebrionidae) from the Mongolia–Xinjiang Region in China. Journal of Hebei University S 1: 1–10. https://api.semanticscholar.org/CorpusID:87064198

[B18] Ren GD Ba YB Li HY Niu YP Zhu XC Li Z Shi AM (2016) Coleoptera: Tenebrionidae (I); Fauna Sinica: Insecta; Science Press: Beijing, China Volume 63: 532p.

[B19] Shi AM (2013) Three new species of *Gnaptorina* Reitter (Coleoptera, Tenebrionidae: Blaptini) from Tibet, China. Zootaxa 3637(4): 462–471. 10.11646/zootaxa.3637.4.5 26046211

[B20] Simmons RB Weller SJ (2001) Utility and evolution of cytochrome b in insects. Molecular Phylogenetics and Evolution 20(2): 196–210. 10.1006/mpev.2001.0958 11476629

[B21] Simon C Frati F Bekenbach A Crespi B Liu H Flook P (1994) Evolution, weighting, and phylogenetic utility of mitochondrial gene sequences and a compilation of conserved polymerase chain reaction primers. Annals of the Entomological Society of America 87(6): 651–701. 10.1093/aesa/87.6.651

[B22] Tang XJ Zhang JY Yu YZ Ren GD Jia L (2023) Description of six *Blaps* pupae (Coleoptera:Tenebrionidae: Blaptini) from China. Journal of Asia-Pacific Biodiversity 16(1): 32–38. 10.1016/j.japb.2022.12.006

[B23] Yu YZ Zhang FJ (2004) The biological characters of *Blaps opaca* Reitter (Coleoptera: Tenebrionidae). Journal of Ningxia Agricultural College 1: 5–7+16.

[B24] Yu YZ Zhang JY (2005) The biological characteristics of *Blaps femoralis*. Chinese Bulletin of Entomology 3: 290–294.

[B25] Yu YZ Ren GD Ma F (1993a) Record and narration on eight species of larvae of Blaptini (Coleoptera, Tenebrionidea). Journal of Ningxia Agricultural College S 1: 59–70.

[B26] Yu YZ Ren GD Ma F (1993b) Record and narration on six species pupae in soll of family Tenebrionidae (Coleoptera). Journal of Ningxia Agricultural College S 1: 79–84.

[B27] Yu YZ Ren GD Sun QX (1996) Morphology and genus and species key of common Blaptini larvae in Northern China. Entomological Knowledge 4: 198–203.

[B28] Yu YZ Ren GD Dai JX (1999a) Identification on the pupae of Tenebrionidae(Coleoptera)from North China. Journal of Ningxia University 4: 364–367. https://europepmc.org/article/CBA/330793 [Natural Science Edition]

[B29] Yu YZ Zhang DZ Wang XP (1999b) The larval morphology of five species of the Blaptini-Tribe (Coleoptera: Tenebrionidea). Journal of Ningxia Agricultural College 4: 15–20.

[B30] Yu YZ Zhang DZ Ren GD (2000) Systematic research of the genus *Blaps* Fabricius–Larvae in China (Coleoptera:Tenebrionidae) (Part I). Journal of Hebei University S 1: 94–101. https://api.semanticscholar.org/CorpusID:87757597

[B31] Zhang DZ Yu YZ Ren GD (2000) Systematic research of the genus *Blaps* Fabricius–Larvae in China (Coleoptera:Tenebrionidae) (Part II). Journal of Hebei University S 1: 102–109. https://api.semanticscholar.org/CorpusID:87832720

[B32] Zhang JY Yu YZ Jia L (2005) Biological characteristic of *Blaps kiritshenkoi* (Coleoptera:Tenebrionidae). Plant Protection 4: 45–48. https://api.semanticscholar.org/CorpusID:86907925

[B33] Zhao M Feng Y Chen XM Ji HH (2009) Morphological and biological characteristics of *Blaps rhynchopetera* (Coleoptera: Tenebrionidea). Journal of Environmental Entomology 31(4): 348–355. https://api.semanticscholar.org/CorpusID:88046216

[B34] Zhu XC Ren GD (2014) The larvae of *Gnaptorinafelicitana* and *Agnaptoriaamdoensis* of the tribe Blaptini from China (Coleoptera: Tenebrionidae). Zoological Systematics 39(02): 275–282.

