# Supplementary material for: ﻿The adult, pupa, and larva of a new species of Gnaptorina Reitter, 1887 (Coleoptera, Tenebrionidae, Blaptini) from the Tibetan Plateau, with molecular phylogenetic inferences
Source: Zookeys. 2024 Jan 23;1190:91–106. doi: 10.3897/zookeys.1190.113126 (PMC10829047; doi:10.3897/zookeys.1190.113126)
Supplement: Supplementary material 1 — List of specimens used in this study with the corresponding accession number [file zookeys-1190-091_article-113126__-s001.docx]

**Table S1.** List of specimens used in this study with the corresponding accession number.

| N0. | Species / Subspecies | Sampling locality | Elevation (m) | Date of collection | Collector(s) | Preservation | Accession numbers  COI / Cytb / 16S / 28S |
| --- | --- | --- | --- | --- | --- | --- | --- |
| 1 | *Gnaptorina kashkarovi* | Haizi Shan, Batang County, Sichuan, China (SCBT) | 4448 | 13.VIII.2016 | X. Li *et al*. | Ethanol | OR856438 / MK415073/ OR856428 / OR856416 |
| 2 | *Gnaptorina kashkarovi* | Cunge, Litang County, Sichuan, China (SCLT) | 4412 | 14.VIII.2016 | X. Li *et al*. | Ethanol | MK415213 / MK415074 MK415122 / OR856416 |
| 3 | *Gnaptorina kashkarovi* | Barong, Baiyü County, Sichuan, China (SCBY) | 3824 | 5.VIII.2016 | X. Li *et al*. | Ethanol | MK415214 / MK415075 MK415123 / MK415188 |
| 4 | *Gnaptorina montana* | Yulong, Jomda County, Xizang, China (XZJD) | 4164 | 9.Ⅷ.2016 | X. Li *et al*. | Ethanol | OR859956 / ––––––––  OR879118 / OR879177 |
| 5 | *Gnaptorina montana* | Namsai, Zadoi County, Qinghai, China (QHZD) | 4045 | 22. Ⅶ.2012 | G. Ren *et al*. | Ethanol | OR859957 / ––––––––  OR879119 / OR879178 |
| 6 | *Gnaptorina montana* | Karmargo, Riwoqê County, Xizang, China (XZLWQ) | 4359 | 22.Ⅷ.2018 | G. Ren *et al*. | Ethanol | OR859958 / ––––––––  OR879120 / OR879179 |
| 7 | *Gnaptorina fairmairei* | Anhong, Songpan County, Sichuan, China (SCSP) | 2710 | 28.VII.2016 | X. Li *et al*. | Ethanol | MK415212 / MK415077 MK415120 / MK415194 |
| 8 | *Gnaptorina fairmairei* | Minjiang, Songpan County, Sichuan, China (SCSP) | 2490 | 28.VII.2016 | X. Li *et al*. | Ethanol | OR856439 / MK415078 OR856429 /MK415195 |
| 9 | *Gnaptorina rufipes* | Galeng, Xunhua County, Qinghai, China (QHXH) | 2627 | 2.VIII.2012 | G. Ren *et al*. | Dried | MK415229 / MK415076 MK415121 / –––––––– |
| 10 | *Gnaptorina kozlovi* | Gyêgu, Yushu County, Qinghai, China (QHYS) | 4008 | 24.Ⅶ.2019 | G. Ren *et al*. | Ethanol | OR859959 / OR891753  OR879121 / OR879180 |
| 11 | *Gnaptorina kozlovi* | Gyêgu, Yushu County, Qinghai, China (QHYS) | 3584 | 12.Ⅷ.2019 | G. Ren *et al*. | Ethanol | OR859960 / OR891754  OR879122 / OR879181 |
| 12 | *Gnaptorina kozlovi* | Baitang, Yushu County, Qing hai, China (QHYSBT) | 4287 | 12.Ⅷ.2019 | G. Ren *et al*. | Ethanol | OR859961 / OR891755  OR879123 / OR879182 |
| 13 | *Gnaptorina kozlovi* | Zhenqin, Chindu County, Qing hai, China (QHCD) | 4394 | 12.Ⅷ.2019 | G. Ren *et al*. | Ethanol | OR859962 / OR891756  OR879124 / OR879183 |
| 14 | *Gnaptorina minxiana* | Dianzi, Lintan, Gansu, China (GSLT) | 2760 | 5.VIII.2009 | G. Ren *et al*. | Dried | MK415231 / –––––––– MK415130 / MK415191 |
| 15 | *Gnaptorina lii* | Fengtai Forest Farm, Longde County, Ningxia, China (NXLD) | 2211 | 14.VII.2014 | L. Bai *et al*. | Ethanol | MK415211 / MK415081 MK415118 / MK415190 |
| 16 | *Gnaptorina crenata* | Baiya, Dêgê County, Sichuan, China (SCDG) | 3021 | 6. VIII.2016 | X. Li *et al*. | Ethanol | MK415158 / –––––––– MK415141 / –––––––– |
| 17 | *Gnaptorina crenata* | Jangra Dêgê County , Sichuan, China (SCDG) | 3120 | 6. VIII.2016 | X. Li *et al*. | Ethanol | MK415157 / MK415168 MK415140 / MK415150 |
| 18 | *Gnaptorina crenata* | Gamtog, Jomda County, Xizang, China (3XZJD) | 3223 | 7. VIII.2016 | X. Li *et al*. | Ethanol | MK415159 / MK415166 MK415142 / MK415151 |
| 19 | *Gnaptorina crenata* | Hepo, Baiyü, Sichuan, China (SCBY) | 3000 | 6.VIII.2016 | X. Li *et al*. | Ethanol | MK415161 / MK415167 MK415145 / MK415152 |
| 20 | *Gnaptorina felicitana* | Sahuteng, Zadoi County, Qinghai, China (QHZD) | 4292 | 22.VII.2012 | G. Ren *et al*. | Dried | OR856445 / MK423896 MK415114 / MK415183 |
| 21 | *Gnaptorina felicitana* | Madoi County, Qinghai, China (QHMD) | 4267 | 16.VII.2009 | G. Ren *et al*. | Dried | MG993069 / MG993079 OR856430 / MG993056 |
| 22 | *Gnaptorina felicitana* | Yiniu, Sêrxü County, Sichuan, China (SCSQ) | 3937 | 18.VII.2009 | G. Ren *et al*. | Dried | MK415207 / MK423895  –––––––– / MK415192 |
| 23 | *Gnaptorina proxima* | Huangcheng, Menyuan, Qinghai, China (QHMY) | 3158 | 30.VII.2012 | G. Ren *et al*. | Dried | MK415232 / MK415087  MK415115 / –––––––– |
| 24 | *Gnaptorina proxima* | Huangcheng, Menyuan County, Qinghai, China (QHYM) |  | 13.Ⅷ.2019 | Z. Zhou *et al*. | Ethanol | [OR863761](https://www.ncbi.nlm.nih.gov/nuccore/OR863761) / ––––––––  [[OR863760](https://www.ncbi.nlm.nih.gov/nuccore/OR863761)](https://www.ncbi.nlm.nih.gov/nuccore/OR863761) / –––––––– |
| 25 | *Gnaptorina media* | Gyêgu, Yushu County, Qinghai, China (QHYS) | 3619 | 20.VII.2012 | G. Ren *et al*. | Dried | MK415208 / MK415079 MK415116 / MK415193 |
| 26 | *Gnaptorina rugosipensis* | Chowa, Dêgê, Sichuan, China (SCDG) | 4007 | 24.VIII.2018 | X. Bai *et al*. | Ethanol | MK423899 / MK415088 MK415117 / –––––––– |
| 27 | *Gnaptorina platytarsia* | Jamda, Jomda County, Xizang, China (XZJD) | 3630 | 8.VIII.2016 | X. Li *et al*.. | Ethanol | MK415209 / MK415080 MK415125 / MK415189 |
| 28 | *Gnaptorina platytarsia* | Tangpu, Jomda County, Xizang, China (XZJD) | 3293 | 6.VIII.2016 | X. Li *et al*. | Ethanol | MK415210 / –––––––– /MK415124 / OR856418 |
| 29 | *Gnaptorina miroshnikovi* | Chola Shan, Dêgê County, Sichuan, China (SCDG) | 4158 | 24.VIII.2018 | X. Bai *et al*. | Ethanol | MK423898 / –––––––– MK415126 / –––––––– |
| 30 | *Gnaptorina crassitibia* | Ronggai, Baiyü County, Sichuan, China (SCBY) | 3120 | 5.Ⅷ.2016 | X. Li *et al.* | Ethanol | OR859963 / OR891757  –––––––– / –––––––– |
| 31 | *Gnaptorina crassitibia* | Ronggai, Baiyü Xian, Sichuan, China (SCBY) | 3120 | 5.Ⅷ.2016 | X. Li *et al.* | Ethanol | –––––––– / OR891758  OR879125 / OR879184 |
| 32 | *Gnaptorina potanini* | Cêruma, Maqu County, Gansu, China (GSMQ) | 3424 | 11.VII.2009 | G. Ren *et al*. | Dried | MK423897 / –––––––– MK415119 / –––––––– |
| 33 | *Gnaptorina lhorongica* | Dawengdang Shan, Lhorong County, Xizang, China (XZLLDWLS) | 3854 | 11.Ⅷ.2015 | G. Ren *et al*. | Ethanol | –––––––– / OR891767  OR879135 / OR879193 |
| 34 | *Gnaptorina lhorongica* | Zhongyi, Lhorong County, Xizang, China (XZLLZY) | 4138 | 30.Ⅶ.2019 | G. Ren *et al*. | Ethanol | OR859971 / OR891768  OR879137 / OR879194 |
| 35 | *Gnaptorina lhorongica* | Zhongyi, Lhorong County, Xizang, China (XZLLZT) | 4138 | 30.Ⅶ.2019 | G. Ren *et al*. | Ethanol | OR859972 / OR891769  OR879136 / OR879195 |
| 36 | *Gnaptorina lhorongica*  larva | Nagjog, Lhorong County, Xizang, China (XZLLLJ) larva | 4680 | 30.Ⅶ.2019 | G. Ren *et al*. | Ethanol | OR859974 / OR891771  OR879139 / OR879198 |
| 37 | *Gnaptorina lhorongica+* pupa | Nagjog, Lhorong County, Xizang, China (XZLLLJ) | 4680 | 30.Ⅶ.2019 | G. Ren *et al*. | Ethanol | OR859975 / OR891772  OR879140 / OR879197 |
| 38 | *Gnaptorina lhorongica* | Nagjog, Lhorong County, Xizang, China (XZLLLJ) | 4680 | 30. VIII.2019 | G. Ren *et al*. | Ethanol | OR859975 / OR891770  OR879138 / OR879196 |
| 39 | *Gnaptorina dongdashanensis* | Dongda Shan, Zogang, Xizang, China (XZZG) | 4621 | 11.VIII.2016 | X. Li *et al*. | Ethanol | MK415205 / MK415082 MK415127 / MK415196 |
| 40 | *Gnaptorina dongdashanensis* | Dongda Shan, Zogang County, Xizang, China (XZZG) | 4787 | 11.VIII.2016 | X.Li *et al*. | Ethanol | MK415206 / MK415083  MK415128 / OR856419 |
| 41 | *Gnaptorina artipennis* | Jinhe, Yanyuan County, Sichuan, China (SCYY) | 1337 | 2.VIII.2015 | G. Ren *et al*. | Ethanol | MK415155 / MK415169 MK415143 / MK415153 |
| 42 | *Gnaptorina artipennis* | Badi, Danba County, Sichuan, China (SCDB) | 2230 | 18.VII.2008 | G. Ren *et al*. | Dried | MK415156 / –––––––– MK415147 / OR856420 |
| 43 | *Gnaptorina medvedevi* | Nyangbo, Gongbo’gyamda County, Xizang, China (XZGBJD) | 4011 | 8.VIII.2019 | G. Ren *et al*. | Ethanol | OR859964 / OR891765  OR879126 / –––––––– |
| 44 | *Gnaptorina longicornis* | Ranwu, Baxoi County, Xizang, China (XZBX) | 4000 | 14.VII.2008 | G. Ren *et al*. | Dried | OR856440 / MK415084 MK415096 / OR856422 |
| 45 | *Gnaptorina longicornis* | Ranwu, Baxoi County, Xizang, China (XZBX) | 4000 | 14.VII.2008 | G. Ren *et al*. | Dried | MK415204 / MK415085 MK415129 / OR856421 |
| 46 | *Gnaptorina longicornis* | Guyu, Zayü County, Xizang, China (XZCY) | 3480 | 31.VII.2017 | X. Bai *et al*. | Ethanol | MK415203 / MK415086 MK415097 / MK415184 |
| 47 | *Gnaptorina polita* | Quzika, Markam County, Xizang, China (XZMK) | 2559 | 8.VIII.2015 | G. Ren *et al*. | Ethanol | MK415154 / MK415170 MK415138 / –––––––– |
| 48 | *Gnaptorina nigera* | Songduoke, Comai County, Xizang, China (XZCM) | 4727 | 8.VIII.2014 | G. Ren *et al*. | Ethanol | MK415216 / MK423893 MK415098 / MK415181 |
| 49 | *Gnaptorina nigera* | Yangjingxue, Damxung County, Xizang, China (XZDX) | 4332 | 23.VII.2014 | G. Ren *et al*. | Ethanol | MG993070 / OR891774  OR856431 / MG993057 |
| 50 | *Gnaptorina nigera* | Yangbajing, Damxung, Xizang, China (XZYBJ) | 4293 | 8.VI.2016 | B.Wang | Ethanol | OR856443 / OR891775 OR856432 / OR856423 |
| 51 | *Gnaptorina nigera* | Damxung, Xizang, China (XZXD) | 4293 | 8.VI.2016 | B.Wang | Ethanol | OR856444 / MG993080 MK415099 / OR856424 |
| 52 | *Gnaptorina nigera* | Shenla, Nagarzê County, Xizang, China (XZLKZ) | 4605 | 6.VIII.2014 | G. Ren *et al*. | Ethanol | MK415216 / MK423894 OR856433 / OR856425 |
| 53 | *Gnaptorina globithoracalis* | Xiangmao, Nagqu County, Xizang, China (XZNQ) | 4605 | 15.VIII.2015 | G. Ren *et al*. | Ethanol | MK415217 / MK415064 MK415100 / MK415182 |
| 54 | *Gnaptorina globithoracalis* | Pubu, Baingoin County, Xizang, China (XZBGPB) | 4727 | 18.VIII.2018 | X. Bai *et al*. | Ethanol | OR859965 / OR891759  OR879127 / OR879186 |
| 55 | *Gnaptorina globithoracalis* | Beila, Baingoin County, Xizang, China (XZBGBL) | 4635 | 3.VIII.2019 | G. Ren *et al*. | Ethanol | OR859966 / OR891760  OR879128 / OR879187 |
| 56 | *Gnaptorina cordicollis* | Qumahe, Qumarlêb County, Qinghai, China (QHQML) | 4325 | 24.VII.2012 | G. Ren *et al*. | Dried | MK415228 / MK415065 MK415101 / MK415199 |
| 57 | *Gnaptorina cordicollis* | Nêbxi, Baqên County, Xizang, China (QHZD) | 4209 | 21.VIII.2018 | X. Bai *et al*. | Ethanol | OR859969 / ––––––––  OR879129 / OR879188 |
| 58 | *Gnaptorina cordicollis* | Pointa, Baqên County, Xizang, China (XZBQBT) | 4367 | 19.VIII.2018 | X. Bai *et al*. | Ethanol | –––––––– / OR891762  OR879130 / OR879189 |
| 59 | *Gnaptorina cordicollis* | Ju’nyung, Sêrxü County, Sichuan, China (SCSQ) | 3937 | 18.Ⅶ.2019 | G. Ren *et al*. | Ethanol | OR859968 / OR891763  OR879133 / –––––––– |
| 60 | *Gnaptorina cordicollis* | Sahuteng, Zadoi County, Qinghai, China (QHZD) | 4092 | 22.Ⅶ.2012 | G. Ren *et al*. | Ethanol | –––––––– / OR891761  OR879132 / OR879190 |
| 61 | *Gnaptorina cordicollis* | Riwoqê County, Xizang, China (XZLWQ) | 3918 | 27.Ⅶ.2019 | G. Ren *et al*. | Ethanol | OR859970 / OR891764  OR879131 / OR879191 |
| 62 | *Gnaptorina ampliptera* | Jinsha, Baiyü County, Sichuan, China (SCBY) | 2980 | 6.VIII.2016 | X. Li *et al*. | Ethanol | –––––––– / MK415164 OR856434 / MK415148 |
| 63 | *Gnaptorina ampliptera* | Baiya, Dêgê County, Sichuan, China (SCDG) | 3021 | 6.VIII.2016 | X. Li *et al*. | Ethanol | MK415163 / MK415165 MK415139 / MK415149 |
| 64 | *Gnaptorina pilifera* | Jianglong, Lhünzê County, Xizang, China (XZLZ) | 4493 | 9.VIII.2014 | G. Ren *et al*. | Ethanol | OR856441 / MK423892 MK415102 / MK415186 |
| 65 | *Gnaptorina pilifera* | Ruogulang, Qusum County, Xizang, China (XZQS) | 4513 | 9.VIII.2014 | G. Ren *et al*. | Ethanol | MK415215 / OR891773 MK415104 / MK415185 |
| 66 | *Gnaptorina pilifera* | Sewu, Qusum County, Xizang, China (XZQS) | 4433 | 9.VIII.2014 | G. Ren *et al*. | Ethanol | MG993071 / MG993081 MK415103 / MK418187 |
| 67 | *Gnaptorina tishkovi* | Rongxar, Tingri County, Xizang, China (XZDR) | 4907 | 26.VII.2014 | G. Ren *et al*. | Ethanol | MG993072 / ––––––––  –––––––– / MG993059 |
| 68 | *Gnaptorina tishkovi* | Rongxar, Tingri County, Xizang, China (XZDR) | 4907 | 26.VII.2014 | G. Ren *et al*. | Ethanol | –––––––MG993082 MK415105 / MG415175 |
| 69 | *Gnaptorina tishkovi* | Qiangbu, Dinggyê County, Xizang, China (XZDJ) | 4698 | 4.VIII.2014 | G. Ren *et al*. | Ethanol | MK415218 / –––––––– MK415107 / MK415176 |
| 70 | *Gnaptorina tishkovi* | Ni La Shan, Dinggyê County, Xizang, China (XZDJ) | 4874 | 4.VIII.2014 | G. Ren *et al*. | Ethanol | MK415219 / –––––––– MK415106 / MK415197 |
| 71 | *Gnaptorina tishkovi* | Riwu, Dinggyê County, Xizang, China (XZDJ) | 4561 | 2.VIII.2014 | G. Ren *et al*. | Ethanol | MK415220 / ––––––––  –––––––– / MK415198 |
| 72 | *Gnaptorina compressa* | Tulong, Nyalam County, Xizang, China (XZNLM) | 4478 | 30.VII.2014 | G. Ren *et al*. | Ethanol | OR856442 / OR891776  OR856435 / OR856426 |
| 73 | *Gnaptorina compressa* | Tong La Shankou, Nyalam County, Xizang, China (XZNLM) | 5126 | 30.VII.2014 | G. Ren *et al*. | Ethanol | MK415221 / MK415066 MK415108 / MK415171 |
| 74 | *Gnaptorina compressa* | Qiongga, Gyirong County, Xizang, China (XZJL) | 4474 | 30.VII.2014 | G. Ren *et al*. | Ethanol | MK415222 / MK415067 MK415109 / MK415172 |
| 75 | *Gnaptorina brucei* | Rongxar, Tingri County, Xizang, China (XZDR) | 4817 | 28.VII.2014 | G. Ren *et al*. | Ethanol | MK415223 / MK415068 MK415110 / MK415173 |
| 76 | *Gnaptorina kangmar* | Tüna, Yadong County, Xizang, China (XZYD) | 4510 | 19.VIII.2015 | G. Ren *et al*. | Ethanol | –––––––– / MK415069 OR856437 / –––––––– |
| 77 | *Gnaptorina kangmar* | Naiqinkangsang Xueshan, Xizang, China (XZJZ) | 5030 | 6.VIII.2014 | G. Ren *et al*. | Ethanol | MK415226 / –––––––– MK415111 / MK415177 |
| 78 | *Gnaptorina kangmar* | Puma Yumco Lake, Nagarzê, Xizang, China (XZLKZ) | 4980 | 6.VIII.2014 | G. Ren *et al*. | Ethanol | MK415230 / MK415071  –––––––– / MK415178 |
| 79 | *Gnaptorina himalaya* | Lhünzê, Xizang, China (XZLZ) | 3872 | 27.VI.2004 | A. Shi *et al*. | Dried | MK415227 / MK415072 MK415112 / MK415180 |
| 80 | *Gnaptorina himalaya* | Yalaxiangbu Shan, Shannan County, Xizang, China (XZSN) | 4888 | 6.Ⅵ.2014 | X. Bai *et al*. | Ethanol | OR859967 / OR891766  OR879134 / OR879192 |
| 81 | *Gnaptorina cuonaensis* | Cona, Cona County, Xizang, China (XZCN) | 4599 | 9.VIII.2014 | G. Ren *et al*. | Ethanol | MK415224 / MK415069 OR856436 / OR856427 |
| 82 | *Gnaptorina cuonaensis* | Yangcuo La Shan, Cona County, Xizang, China(XZCN) | 4670 | 9.VIII.2014 | G. Ren *et al*. | Ethanol | MK415225 / MK415070 MK415113 / MK415179 |
| 83 | *Oodescelis affinis* | Tekes County, Xinjiang Aut. Reg., China (XJTK) |  | 21.V.2009 | D. Sun *et al.* | Dried | MG993073 / MG993083 MH410299 / –––––––– |
| 84 | *Oodescelis oblonga* | Kuerdening, Gongliu County, Xinjiang Aut. Reg., China (XJGL) |  | 4.VIII.2007 | C. Zhang *et al.* | Dried | MG993076 / MG993086 MH410298 / –––––––– |
| 85 | *Oodescelis emmerichi* | Niubeiliang, Zhashui County, Shanxi Prov., China (SXZS) |  | 22.VIII.2011 | X. Zhu *et al.* | Dried | MH185099 / MG993084 MH185103 / –––––––– |
| 86 | *Oodescelis punctatissima* | Li Shan, Shanxi Prov., China (SXLS) |  | 21.VII.2013 | X.Zhu *et al.* | Dried | MH185093 / MH194706 MH185106 / –––––––– |
